# Nuclear protein in testis midline carcinoma of nasal cavity: a case report and review of the literature

**DOI:** 10.3389/fonc.2026.1859581

**Published:** 2026-09-29

**Authors:** Kexin Meng, Xiaotong Huang, Haijun Lu

**Affiliations:** 1 Department of Radiation Oncology, The Affiliated Hospital of Qingdao University, Qingdao, China; 2 Medical College, Qingdao University, Qingdao, China

**Keywords:** immunotherapy, multidisciplinary therapy, nasal cavity neoplasm, nuclear protein in testis, NUT midline carcinoma

## Abstract

Nuclear protein in testis (NUT) midline carcinoma (NMC) is an extremely rare, highly aggressive, and poorly differentiated malignant neoplasm that predominantly arises from the midline epithelial structures of the head and neck. It is driven by genetic rearrangements of the NUTM1 gene, with the most common aberration being a translocation that forms a fusion gene with BRD4 on chromosome 19p13.1. The diagnosis of NMC is challenging due to its non-specific clinical and histological features, and it typically requires a combination of immunohistochemical detection of NUT protein and molecular assays to confirm NUTM1 gene rearrangement. The prognosis of NMC is dismal, with a median overall survival of less than one year. To date, no standard treatment regimen has been established, and only individualized therapeutic strategies have been reported in the literature. Herein, we present a case of a 40-year-old female patient with NMC of the left nasal cavity who presented with nasal mass, nasal obstruction, rhinorrhea, mild epistaxis, and hyposmia. The patient was diagnosed with NMC based on pathological and immunohistochemical findings, and was staged as T2N0M0. She received multidisciplinary treatment including neoadjuvant chemotherapy, radical surgery, adjuvant chemoradiotherapy, and subsequent immune checkpoint inhibitor (ICI) maintenance therapy. Up to the latest follow-up in February 2026, the patient had achieved complete remission with no evidence of tumor recurrence. We conclude that comprehensive and intensive first-line multidisciplinary therapy is crucial for improving clinical outcomes in patients with NMC, a disease with inherently high risks of recurrence and progression and a poor natural prognosis.

## Introduction

Nuclear protein in testis (NUT) midline carcinoma (NMC) is a rare and highly aggressive poorly differentiated carcinoma first described in 2002, characterized by recurrent genetic rearrangements of the NUTM1 gene on chromosome 15q14 (1, 2). The tumor predominantly arises from the midline epithelial tissues of the head and neck, including the nasal cavity, paranasal sinuses, pharynx, and larynx, and can also occur in the mediastinum, lung, and other midline structures (3–6). The most common genetic alteration in NMC is the translocation of NUTM1 with BRD4 on chromosome 19p13.1, forming the BRD4-NUTM1 fusion gene, which accounts for approximately 70% of all NMC cases; other rare fusion partners include BRD3 and NSD3 (7, 8).

NMC lacks specific clinical and morphological features, making it easily misdiagnosed as other poorly differentiated carcinomas such as squamous cell carcinoma, adenocarcinoma, or undifferentiated carcinoma in clinical practice (9). The gold standard for diagnosis combines histological evaluation with immunohistochemical staining for NUT protein (nuclear dot-like positive staining) and molecular genetic testing to confirm NUTM1 gene rearrangement (10). However, due to the limited availability of molecular testing in some institutions, positive NUT protein expression by immunohistochemistry, combined with typical clinical and histological features, is sufficient for a tentative diagnosis of NMC (11).

NMC is associated with an extremely poor prognosis, with a median overall survival of less than 12 months in untreated patients and a 5-year overall survival rate of less than 10% (12). To date, there is no universally accepted standard treatment regimen for NMC, and clinical management remains challenging. Current therapeutic strategies are mostly individualized and based on multidisciplinary team (MDT) discussions, including surgery, chemotherapy, radiotherapy, and targeted therapy; immunotherapy has also emerged as a potential therapeutic option in recent years (13, 14). Here, we report a case of NMC of the left nasal cavity in a 40-year-old female who achieved long-term complete remission with a comprehensive multidisciplinary treatment approach, and review the relevant literature to provide insights for the clinical management of this rare malignancy.

## Case presentation

A 40-year-old female presented to our hospital with a left nasal cavity mass accompanied by nasal obstruction, rhinorrhea, intermittent mild epistaxis, and hyposmia. She had no significant past medical history or family history of malignant tumors. A biopsy of the left nasal cavity mass was performed at an external hospital, and the pathological sections were referred to our hospital for consultation. On October 17, 2023, the consultation result of the external pathological sections showed: mucosal erosion of the left nasal vestibule with infiltration of degenerative atypical cells, consistent with malignant neoplasm. Immunohistochemistry (IHC) results: CK (+), P40 (+), NUT (+), Syn (-), CgA (-), S-100 (-), Brg1 (no deletion), CD99 (+), HMB45 (-), INI-1 (no deletion), EBER (*in situ* hybridization, ISH) (-) (including positive control). Combined with IHC findings, NMC was highly suspected. On October 24, 2023, the re-sectioned pathological result of the external paraffin block in our hospital revealed: poorly differentiated malignant neoplasm with significant cellular atypia, necrosis, and focal palisading arrangement. IHC results: CK (weak +), P40 (+), NUT (+), P63 (+), SMA (focal +), Syn (-), CgA (-), Ki-67 (+, 40%), S-100 (-), Brg1 (+), INI-1 (+), CD99 (membranous weak +), MLH1 (+), MSH2 (+), MSH6 (+), PMS2 (+), HER2 (0), EGFR (focal weak +). EBER (ISH) (-). PD-L1 immunohistochemical testing was performed using the Ventana SP263 assay on the Ventana BenchMark Ultra platform, with adequate quality control (both negative and positive controls successful). The proportion of PD-L1-positive tumor cells (TC) was <1%, and the proportion of PD-L1-positive tumor-associated immune cells (IC) was 2%. The Combined Positive Score (CPS) was not assessed in this case. Tumor mutational burden (TMB) was not assessed due to the limited availability of tissue for additional molecular testing. Based on the above IHC findings, the diagnosis of NMC was highly suspected, and genetic testing for the NUTM1 gene at a higher-level hospital was recommended for further confirmation. Genetic testing results revealed BRD4 deletion, HDAC10 amplification, NTRK1 amplification, TSC2 amplification, and PDCD1 amplification.

On November 14, 2023, contrast-enhanced magnetic resonance imaging (MRI) of the paranasal sinuses showed a nodular abnormal signal in the anterior part of the left nasal cavity, specifically involving the left middle nasal meatus with the tumor pedicle attached to the anterosuperior nasal septum. The lesion demonstrated heterogeneous internal signal, ill-defined borders, and rough edges, with close proximity to the left lateral nasal wall and nasal roof. The lesion measured 13 mm × 31 mm × 12 mm (left-right × anterior-posterior × superior-inferior) with heterogeneous enhancement after contrast administration, and ill-defined boundaries with adjacent structures (**Figures 1A–C**). These findings were consistent with malignant neoplasm. Contrast-enhanced MRI of the neck showed no obvious enlarged lymph nodes in the bilateral neck. Based on the above findings, the clinical stage of the tumor was determined as cT2N0M0 according to the 8th edition of the American Joint Committee on Cancer (AJCC) staging system.

**Figure 1:**
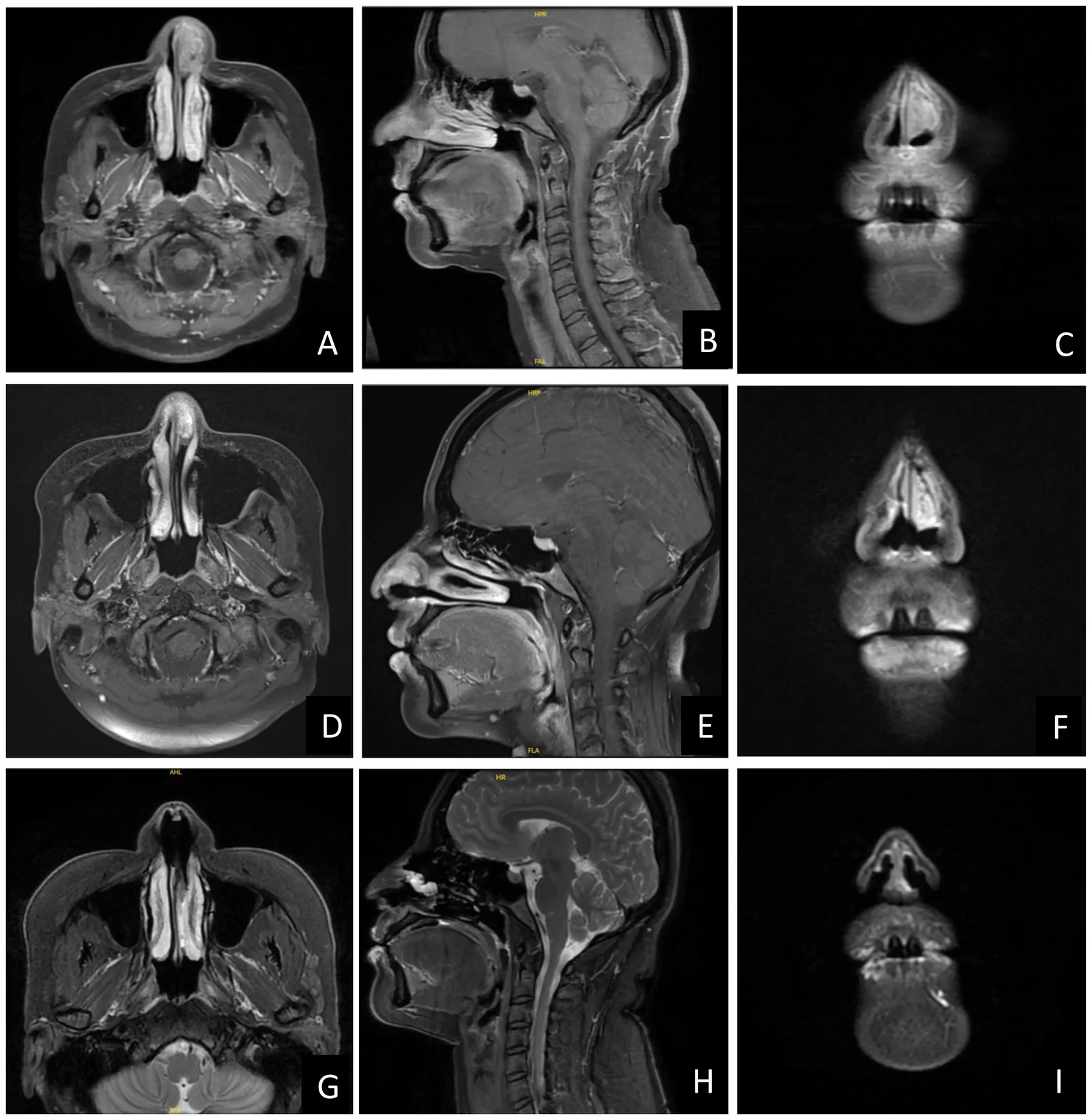
Imaging of the tumor. **(A–C)** Contrast-enhanced MRI of the nasal cavity and paranasal sinuses on Nov.14, 2023 showed a mass in the anterior part of the left nasal cavity measuring 13mm*31mm*12mm. **(A)** for axial, **(B)** for sagittal, and **(C)** for coronal views. **(D–F)** Contrast-enhanced MRI of the nasal cavity and paranasal sinuses on Dec.18, 2023 showed a mass in the anterior part of the left nasal cavity measuring 13mm*23mm*12mm. **(D)** for axial, **(E)** for sagittal, and **(F)** for coronal views. **(G–I)** MRI of the nasal cavity and paranasal sinuses on Dec.30, 2023 showed postoperative findings. **(G)** for axial, **(H)** for sagittal, and **(I)** for coronal views.

After discussion by the hospital’s MDT (consisting of oncologists, otorhinolaryngologists, radiation oncologists, pathologists, and radiologists), the patient was recommended to receive neoadjuvant chemotherapy followed by surgical resection. After excluding related contraindications, the patient received the first two cycles of neoadjuvant chemotherapy with the EP regimen on November 14, 2023, and December 8, 2023, respectively. The regimen consisted of etoposide 0.19 g on days 1–3 plus cisplatin 70 mg on days 1–2. A follow-up contrast-enhanced MRI of the paranasal sinuses on December 18, 2023, showed a nodular heterogeneously enhancing lesion in the anterior left nasal cavity measuring 13 mm × 23 mm × 12 mm (left-right × anterior-posterior × superior-inferior), with a slight local reduction in volume compared with the baseline scan; the borders with adjacent structures remained ill-defined (**Figures 1D–F**). Preoperative electronic fiberoptic laryngoscopy (December 20, 2023) revealed dry nasal mucosa with a hyperplastic mass in the anterior left nasal cavity covered with dry crusts; the bilateral inferior turbinates were not hypertrophied, no neoplasms or purulent secretions were observed in the bilateral middle nasal meatus or olfactory clefts, and the nasal septum was essentially midline. The nasopharynx, oropharynx, hypopharynx, and larynx were all normal, with no evidence of tumor extension beyond the left nasal cavity (**Figures 2A–C**). The efficacy of the two cycles of neoadjuvant chemotherapy was evaluated as Stable Disease (SD) according to the Response Evaluation Criteria in Solid Tumors (RECIST) v1.1.

**Figure 2:**
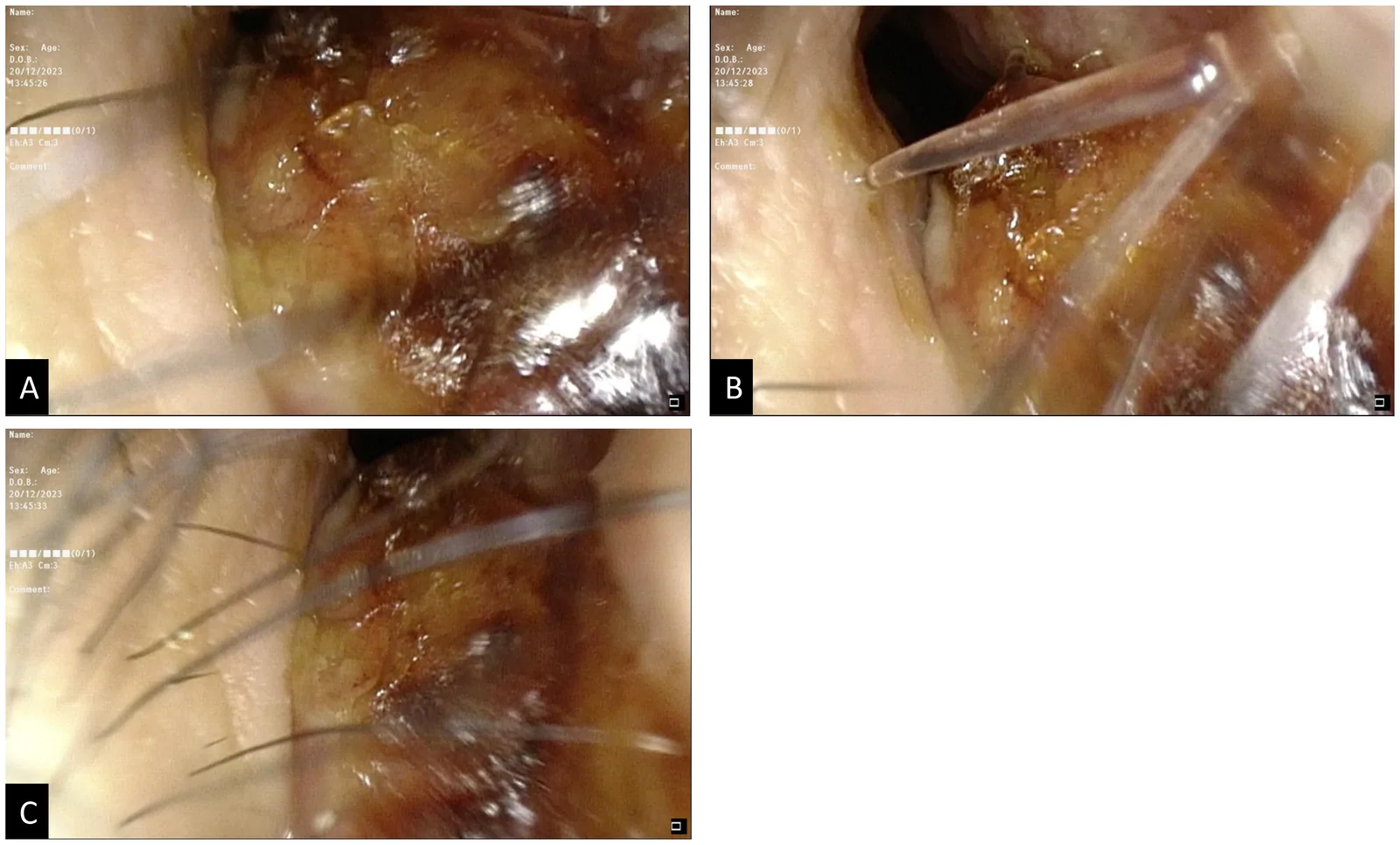
Imaging of the tumor. **(A–C)** Contrast-enhanced MRI of the nasal cavity and paranasal sinuses on Nov.14, 2023 showed a mass in the anterior part of the left nasal cavity measuring 13mm*31mm*12mm. **(A)** for axial, **(B)** for sagittal, and **(C)** for coronal views.

After excluding surgical contraindications, the patient underwent endoscopic endonasal resection of the left nasal cavity tumor under general anesthesia with orotracheal intubation on December 22, 2023. After nasal surface anesthesia with 1% lidocaine cotton pads (1% lidocaine 40 mL + 0.1% epinephrine 2 mL) and local infiltration anesthesia, an arcuate incision was made along the medial mucosa of the nasal vestibule with a small scalpel. The left mucoperichondrium of the nasal septum was incised and elevated posteriorly to the tumor. Intraoperative exploration revealed a mulberry-like, friable mass in the left middle nasal meatus with a pedicle attached to the anterosuperior nasal septum. The tumor was resected en bloc with its pedicle and the surrounding nasal septal mucosa. Subsequently, the mucosa of the nasal columella anterior to the tumor was resected to normal tissue, and the mucosa of the superior nasal septum was resected laterally to the lateral nasal wall. Thus, a portion of the anterosuperior nasal septal mucosa (with the tumor pedicle) was removed, while the cartilaginous and bony framework of the nasal septum was preserved. Intraoperative frozen section pathology showed cancer involvement in the posterosuperior, anteroinferior, anterosuperior, deep anteromedial, posteroinferior, and superomedial margins, with no cancer involvement in the inferoanterior, posteromedial, posteroinferior, and posterosuperior margins. Therefore, further extended resection of the tumor bed and surrounding mucosa was performed. The operation was successful, with an estimated intraoperative blood loss of approximately 10 mL. Postoperative MRI of the nasal cavity and paranasal sinuses showed no sign of residual tumor (**Figures 1G–I**). The final postoperative pathological result showed poorly differentiated carcinoma in the left nasal cavity mass, left nasal columella mass, and left nasal roof mass, with cancer infiltration in the fibrous tissue of the left lateral nasal wall and roof. Postoperative IHC results: CK (+), P40 (+), NUT (+), Syn (-), S-100 (focal +), Brg1 (+, no deletion), CD99 (+), HMB45 (-), INI-1 (+), CgA (-). EBER (ISH) (-) (including positive control). Combined with the clinical history and IHC findings, the diagnosis of NMC was confirmed.

Given the presence of high-risk postoperative factors (positive surgical margins on final pathology), adjuvant chemoradiotherapy was recommended. The EP (etoposide + cisplatin) regimen was selected based on the germ cell tumor-like molecular characteristics of NMC (involvement of the testis-specific NUTM1 gene) and the demonstrated activity of topoisomerase II inhibitors against NMC in preclinical drug screens; cisplatin (DNA cross-linking) combined with etoposide (topoisomerase II inhibition) provides synergistic DNA-damage-driven apoptosis. The third cycle of adjuvant chemotherapy with the EP regimen (etoposide 0.19 g on days 1–3 plus cisplatin 70 mg on days 1–2) was administered on December 29, 2023. Helical Tomotherapy (TOMO) was administered from January 18 to March 5, 2024. The radiotherapy doses were as follows: PGTV 6600 cGy in 30 fractions (220 cGy/fraction), PTV 6000 cGy in 30 fractions (200 cGy/fraction). During radiotherapy, two cycles of concurrent single-agent cisplatin chemotherapy were given on January 22, 2024, and March 1, 2024, with cisplatin 60 mg on days 1–2 per cycle. The 4th and 5th cycles of adjuvant EP chemotherapy were administered on April 15, 2024, and May 20, 2024, respectively, with a reduced dose (etoposide 0.16 g on days 1–3 plus cisplatin 60 mg on days 1–2 per cycle) to improve tolerability after the cumulative toxicity of surgery and chemoradiotherapy. This sandwich approach, as adjuvant EP before concurrent chemoradiotherapy followed by consolidation EP after radiotherapy, was designed to provide maximal systemic and locoregional control for this highly aggressive malignancy with positive surgical margins.

Following the completion of adjuvant chemoradiotherapy, the patient initiated maintenance therapy with cadonilimab, a dual PD-1/CTLA-4 bispecific antibody, at a dose of 375 mg every 3 weeks. The rationale for ICI maintenance was based on the patient’s PD-L1 expression (IC 2%), PDCD1 gene amplification on genetic testing, and the potential synergistic effect of radiotherapy with subsequent immunotherapy (radiotherapy-induced immunogenic cell death). As of the latest follow-up in February 2026, the patient had received a total of 30 cycles of cadonilimab maintenance therapy and continued treatment with no evidence of tumor recurrence or metastasis on regular imaging and clinical evaluations (**Figure 3**). Throughout the 30 cycles of maintenance therapy, the patient did not experience any immune-related adverse events (irAEs). Complete blood counts and liver and renal function tests remained within normal limits, and no skin rash or other immune-mediated toxicities were observed. The patient continues to report mild nasal dryness and discomfort, which is attributed to postoperative and radiotherapy-related mucosal changes rather than irAEs; regular nasal endoscopy demonstrated crusting and yellowish discharge in the nasal cavity, which was managed with routine nasal debridement and irrigation.

**Figure 3:**
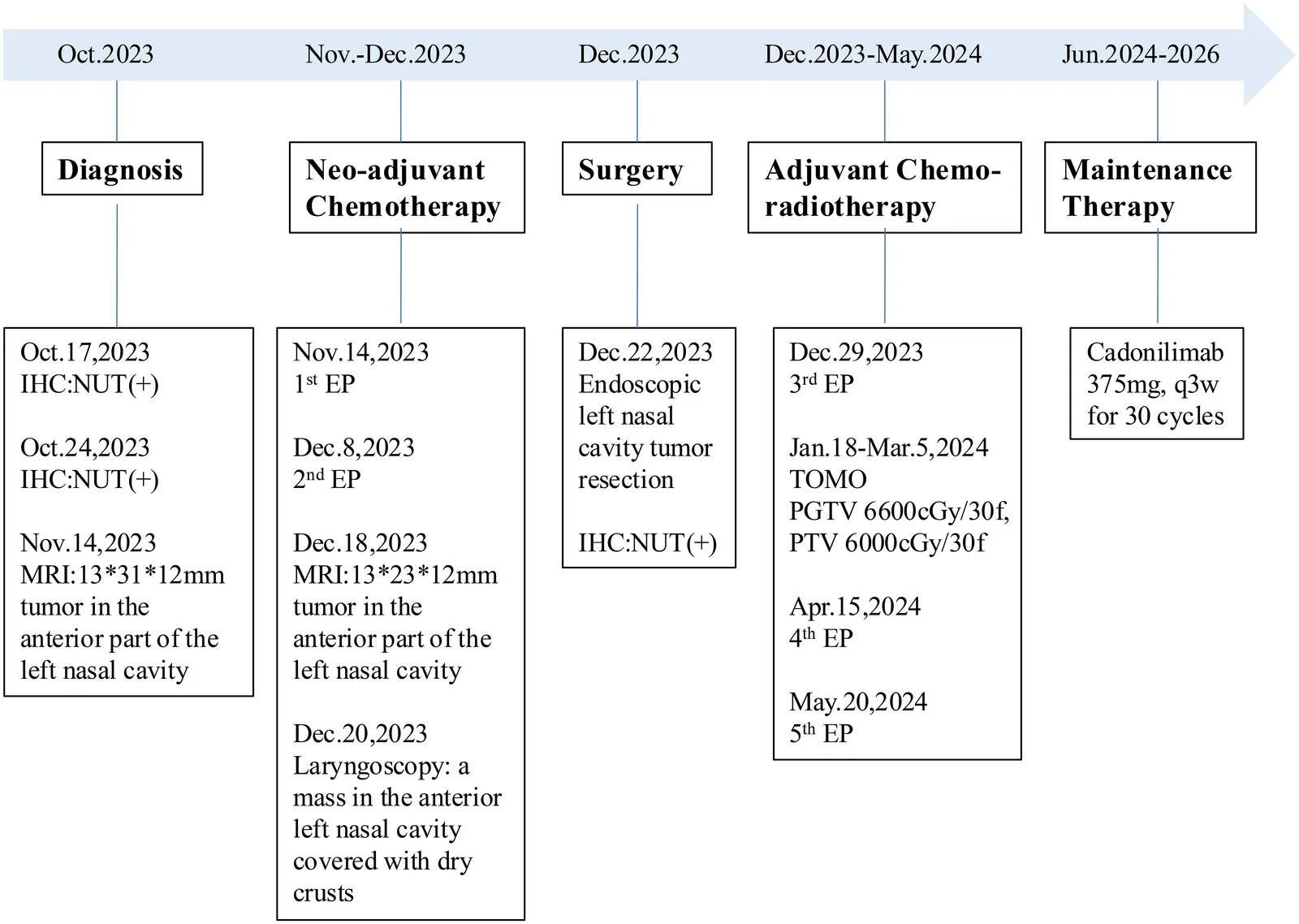
Timeline showcasing relevant data from the episode of care. IHC, Immunohistochemistry; (+), positive expression; MRI, magnetic resonance imaging; EP, chemotherapy of etoposide and cisplatin; TOMO, TOMO radiotherapy; PGTV, planning gross tumor volume; PTV, planning target volume.

### Patient perspective

The patient reported that the initial diagnosis of a rare and aggressive malignancy caused significant anxiety and uncertainty, particularly given the limited available treatment options and poor prognosis described in the literature. During neoadjuvant chemotherapy, she experienced mild fatigue and nausea, which were well managed with supportive care and did not require dose modification. After endoscopic surgery, she reported nasal dryness, mild pain, and intermittent crusting, which gradually improved with regular nasal irrigation and debridement. During concurrent chemoradiotherapy, she experienced taste changes and mild oral mucositis, which resolved within several weeks after completing radiotherapy. She reported good adherence to the cadonilimab maintenance schedule, attending every 3-week infusion without delay, and expressed satisfaction with the tolerability of maintenance immunotherapy. As of the latest follow-up, the patient has returned to her normal daily activities and work, and expressed deep gratitude to the multidisciplinary team for the comprehensive care and the favorable treatment outcome. The patient provided written informed consent for the publication of this case report, including her personal perspective.

## Discussion

NMC is an ultra-rare, highly aggressive malignant tumor with a very limited number of reported cases worldwide, and its clinical management remains a major challenge due to the lack of standard treatment guidelines (3, 15, 16). This case report describes a 40-year-old female with NMC of the left nasal cavity who achieved long-term complete remission with a comprehensive multidisciplinary treatment strategy including neoadjuvant chemotherapy, radical endoscopic surgery, adjuvant chemoradiotherapy, and ICI maintenance therapy, providing valuable clinical experience for the treatment of this rare disease.

The diagnosis of NMC is based on a combination of clinical features, histological morphology, IHC staining, and molecular genetic testing (10). Histologically, NMC typically presents as poorly differentiated carcinoma with significant cellular atypia, frequent mitotic figures, and extensive necrosis; focal squamous differentiation may be observed in some cases, which can lead to misdiagnosis as squamous cell carcinoma (17, 18). The hallmark of NMC diagnosis is the detection of NUTM1 gene rearrangement, with IHC staining for NUT protein serving as a rapid and reliable screening method—nuclear dot-like positive staining of NUT protein is highly specific for NMC, with a specificity of nearly 100%. In this case, the patient had typical clinical manifestations of nasal cavity NMC, and NUT protein was consistently positive on multiple IHC assays reviewed by senior pathologists from multiple tertiary hospitals, meeting the clinical diagnostic criteria for NMC despite the absence of detected NUTM1 fusion gene by genetic testing. The possible reasons for the negative NUTM1 fusion gene test include the presence of rare or novel fusion partners of NUTM1 that are not covered by the commercial genetic testing panel, or technical limitations of the testing method. This finding also suggests that positive NUT protein expression by IHC can be used as a diagnostic basis for NMC in clinical settings where molecular testing is not available or yields negative results.

To date, no standard treatment regimen has been established for NMC, and multidisciplinary treatment is the mainstay of clinical management (13). Chemotherapy is the core of systemic treatment for NMC, with platinum-based combination regimens being the most commonly used first-line regimens (14). In the present case, the EP (etoposide + cisplatin) regimen was selected based on the following rationale: (1) NMC shares germ cell tumor (GCT)-like molecular characteristics—the involvement of the testis-specific NUTM1 gene suggests potential GCT-like biology, and EP is the standard chemotherapeutic backbone for germ cell tumors; (2) etoposide, a topoisomerase II inhibitor, has demonstrated activity against NMC in preclinical *in vitro* and *in vivo* drug screens; (3) cisplatin (DNA cross-linking) combined with etoposide (topoisomerase II inhibition leading to DNA double-strand breaks) provides synergistic DNA-damage-driven apoptosis, which is particularly effective for rapidly proliferating, poorly differentiated malignancies such as NMC. A recent case report also demonstrated the efficacy of the EP regimen combined with anlotinib in pulmonary NUT carcinoma, further supporting the use of EP-based chemotherapy in this disease. In addition to conventional chemotherapy, BET bromodomain inhibitors targeting the BRD4-NUT fusion protein have shown promising efficacy in preclinical and early clinical studies, and are currently the most studied targeted therapy for NMC (19–21). In this case, the patient received two cycles of neoadjuvant EP chemotherapy, achieving SD, which laid the foundation for subsequent surgical resection.

Surgical treatment is the preferred local treatment for resectable NMC of the head and neck (14), and endoscopic tumor resection is a minimally invasive surgical approach with the advantages of clear surgical field, minimal trauma, and rapid postoperative recovery, which is widely used in the treatment of nasal cavity and paranasal sinus malignancies (22). In this case, the patient underwent endoscopic left nasal cavity tumor resection with extended resection due to positive frozen section margins, which effectively removed the local tumor lesion.

Radiotherapy is an important adjuvant treatment for NMC, especially for patients with positive surgical margins, locally advanced disease, or unresectable tumors (23). Concurrent chemoradiotherapy is recommended for high-risk patients to improve local control rates (24). In this case, the patient received TOMO radiotherapy with concurrent cisplatin chemotherapy due to positive postoperative surgical margins, which is a state-of-the-art radiotherapy technique with the advantages of precise targeting, uniform dose distribution, and minimal damage to surrounding normal tissues, effectively reducing the risk of local recurrence.

Immunotherapy has emerged as a novel therapeutic strategy for advanced solid tumors in recent years, and PD-1/PD-L1 inhibitors have shown promising efficacy in some rare malignancies (25). Accumulating evidence supports the potential role of immune checkpoint inhibitors in NUT carcinoma, particularly as maintenance therapy after radiotherapy. A case report described an 87-year-old patient with metastatic sinonasal NUT carcinoma who achieved sustained remission for over 2 years with palliative radiotherapy followed by pembrolizumab maintenance, demonstrating the feasibility of ICI maintenance after radiotherapy in sinonasal NUT carcinoma even in elderly patients with metastatic disease (26). Another case report documented immunotherapy experience in sinonasal NUT midline carcinoma, and a case of parotid NUT carcinoma achieved disease control with sintilimab combined with lenvatinib and a BET inhibitor (27). In thoracic NUT carcinoma, a case series of six pulmonary NUT carcinoma patients demonstrated diverse responses to immunotherapy, including one patient who achieved pathologic complete response with atezolizumab despite negative PD-L1 expression (28); a literature review of 15 pulmonary NUT carcinoma patients who received ICIs showed a significantly prolonged median survival of 13.8 months compared with 4.4 months in those who did not (29). Another case report described a pulmonary NUT carcinoma patient who achieved an initial partial response to EP chemotherapy combined with sintilimab, although the response was transient (30). A case report showed that immunotherapy combined with conventional treatment can significantly improve the overall survival of NMC patients (31). In this case, PD-L1 testing using the Ventana SP263 assay showed TC <1% and IC 2%; the CPS was not assessed, and TMB was not tested due to limited tissue availability. Despite the low PD-L1 expression, the presence of PDCD1 gene amplification on genetic testing provided an additional molecular rationale for ICI use—previous studies have confirmed that PDCD1 amplification is a predictive biomarker for favorable response to anti-PD-1 therapy in solid tumors. The absence of CPS and TMB assessment is acknowledged as a limitation of this study. The patient received cadonilimab, a novel dual anti-PD-1/CTLA-4 bispecific monoclonal antibody independently developed in China, for maintenance therapy after completing chemoradiotherapy. Through its dual mechanism of action, cadonilimab simultaneously blocks PD-1 and CTLA-4 signaling pathways, achieving more effective T-cell activation within the tumor microenvironment while potentially reducing systemic toxicity compared to the combination of two separate monoclonal antibodies. Cadonilimab has shown promising efficacy in multiple solid tumors (32, 33), including a recent Phase II trial in locally advanced head and neck squamous cell carcinoma demonstrating an objective response rate of 87.5% and a pathologic complete response rate of 50% with manageable toxicities (34). Furthermore, radiotherapy can induce immunogenic cell death and enhance anti-tumor immune responses (the abscopal effect), providing a mechanistic rationale for ICI maintenance after chemoradiotherapy. In the present case, the patient received 30 cycles of cadonilimab maintenance therapy and achieved long-term complete remission with no evidence of recurrence up to the latest follow-up. No immune-related adverse events were observed throughout the 30 cycles of maintenance therapy; complete blood counts, liver and renal function tests remained normal, and no skin rash or other immune-mediated toxicities occurred. The mild nasal dryness and persistent crusting were attributed to postoperative and radiotherapy-related mucosal changes rather than irAEs. This finding suggests that immunotherapy may be a potential effective therapeutic option for NMC, especially as maintenance therapy after first-line chemoradiotherapy, and further clinical studies are needed to validate its efficacy and safety.

NMC is associated with an extremely poor natural prognosis, with a median overall survival of less than one year (3). However, accumulating evidence suggests that comprehensive and intensive multidisciplinary treatment can significantly improve the clinical outcomes of NMC patients (12). In this case, the patient achieved long-term complete remission with a combination of neoadjuvant chemotherapy, radical surgery, adjuvant chemoradiotherapy, and immunotherapy maintenance, which highlights the importance of early diagnosis and comprehensive multidisciplinary treatment for NMC. Hopefully, with the development of precision oncology, more molecular targets will be identified, which may further improve the prognosis of NMC patients with relevant genetic alterations.

The World Health Organization (WHO) has included NUT carcinoma as a separate pathological entity in its 2022 Classification of Tumors of the Head and Neck (35), and all major international authorities recommend multidisciplinary treatment as the standard of care. However, due to the rarity of NMC, most treatment recommendations are based on retrospective studies and case reports, and there is still an urgent need for multi-center, large-sample clinical studies to establish standardized treatment regimens.

## Conclusion

NMC is an extremely rare, highly aggressive poorly differentiated carcinoma with no established standard treatment regimen and a dismal prognosis. We report a case of nasal cavity NMC in a 40-year-old female who achieved long-term complete remission with a comprehensive multidisciplinary treatment approach including neoadjuvant EP chemotherapy, endoscopic radical tumor resection, adjuvant chemoradiotherapy, and cadonilimab immunotherapy maintenance. This case suggests that comprehensive and intensive first-line multidisciplinary therapy is crucial for improving the clinical outcomes of NMC patients, and immunotherapy may serve as an effective maintenance treatment option for selected patients with relevant molecular features. Given the rarity of NMC, multi-center, large-sample clinical studies are urgently needed to further explore the optimal diagnostic methods and therapeutic strategies for this rare malignancy, and to identify potential molecular targets for targeted therapy to further improve the prognosis of NMC patients.

## Data Availability

The raw data supporting the conclusions of this article will be made available by the authors, without undue reservation.
